# Leukemic stem cell signatures identify novel therapeutics targeting acute myeloid leukemia

**DOI:** 10.1038/s41408-018-0087-2

**Published:** 2018-06-06

**Authors:** Isabelle Laverdière, Meaghan Boileau, Andrea L. Neumann, Héloïse Frison, Amanda Mitchell, Stanley W. K. Ng, Jean C. Y. Wang, Mark D. Minden, Kolja Eppert

**Affiliations:** 1 Department of Pediatrics, McGill University, Montreal, QC Canada; 2 Research Institute of the McGill University Health Centre, Montreal, QC Canada; 3 Division of Experimental Medicine, Department of Medicine, McGill University, Montreal, QC Canada; 4 Princess Margaret Cancer Centre, University Health Network, Toronto, ON Canada; 5 Department of Chemical Engineering and Applied Chemistry, Institute of Biomaterials and Biomedical Engineering and University of Toronto, Toronto, ON Canada; 6 Division of Medical Oncology and Hematology, Department of Medicine, University of Toronto, Toronto, ON Canada

**Keywords:** Cancer stem cells, Cancer therapeutic resistance

## Abstract

Therapy for acute myeloid leukemia (AML) involves intense cytotoxic treatment and yet approximately 70% of AML are refractory to initial therapy or eventually relapse. This is at least partially driven by the chemo-resistant nature of the leukemic stem cells (LSCs) that sustain the disease, and therefore novel anti-LSC therapies could decrease relapses and improve survival. We performed in silico analysis of highly prognostic human AML LSC gene expression signatures using existing datasets of drug–gene interactions to identify compounds predicted to target LSC gene programs. Filtering against compounds that would inhibit a hematopoietic stem cell (HSC) gene signature resulted in a list of 151 anti-LSC candidates. Using a novel in vitro LSC assay, we screened 84 candidate compounds at multiple doses and confirmed 14 drugs that effectively eliminate human AML LSCs. Three drug families presenting with multiple hits, namely antihistamines (astemizole and terfenadine), cardiac glycosides (strophanthidin, digoxin and ouabain) and glucocorticoids (budesonide, halcinonide and mometasone), were validated for their activity against human primary AML samples. Our study demonstrates the efficacy of combining computational analysis of stem cell gene expression signatures with in vitro screening to identify novel compounds that target the therapy-resistant LSC at the root of relapse in AML.

## Introduction

For more than four decades, the standard of care for acute myeloid leukemia (AML) has not changed significantly and consists of an intensive combined chemotherapy with cytarabine plus an anthracycline as the cornerstone drugs^1,2^. Despite the achievement of remission for a majority of patients, up to 70% of adults and 30% of children will not survive beyond 5 years after initial clinical response due to relapsing disease^3–5^. This highlights the urgent and unmet need for novel drugs to enable a sustainable recovery in patients with AML. Repurposing of drugs, which consists of using a known drug to treat a new indication, is an approach with high potential for the rapid introduction of new therapeutics into the clinic^6,7^. This strategy exploits the known toxicology and pharmacological properties of approved drugs to accelerate regulatory approval. Repurposing is possible because many drugs target multiple pathways, in addition to those that have been described for their current clinical use. For example, thalidomide, once used to treat morning sickness and withdrawn for triggering phocomelia, has been repurposed as a highly effective treatment for myeloma^8^.

In AML, leukemia stem cells (LSCs) produce all the leukemic cells in the patient and therefore a lasting cure for this disease is dependent on eradication of these cells^9^. However, LSCs are relatively resistant to standard therapies^10–12^. For example, these cells are less sensitive to killing by daunorubicin and cytarabine, partially due to increased expression of multidrug resistance genes (i.e., ABCC1/LRP) and their quiescent state, which reduces the effects of cytotoxic agents that target rapidly replicating cells^12–14^. There have been some examples of success in developing anti-LSC compounds^15–18^. However, in general the current methodologies to identify chemotherapeutic agents effective in AML are based upon readouts of general toxicity in the bulk of leukemic cells, and thereby paradoxically ignore LSCs and their unique features. In addition, LSCs are very similar to their normal hematopoietic stem cell (HSC) counterparts. Like HSCs, they utilize similar molecular mechanisms to self-renew, maintain an undifferentiated state and produce progeny that differentiate into more mature progenitor cells^9^. Thus, the biological parallels between AML and normal hematopoiesis highlight the difficulty of developing compounds that target LSCs for elimination without also eradicating HSCs.

We have identified two gene expression signatures for LSCs from analysis of primary human AML samples that were sorted into LSC and non-LSC fractions and validated by xenotransplantation. The high expression of each of these signatures is tightly linked to poor survival in AML and failure of standard therapy across all AML subtypes^19,20^. In silico analysis of gene expression can be used to identify drug candidates^21,22^. The molecular pathways represented in our gene signatures are therefore targets for the development of novel therapeutics against AML through the elimination of LSCs. Despite the substantial similarity between leukemic and healthy stem cells, we generated an HSC-specific gene expression signature as well^19^. Utilizing both LSC and HSC signatures allows for a strategy for the identification of drugs that target LSCs without harming normal HSCs.

To identify anti-LSC compounds, we probed our LSC and HSC gene expression signatures against an existing database of drug–gene interactions to identify compounds predicted to negatively affect the gene expression program of leukemia while sparing normal hematopoietic function. Candidates identified in the in silico analysis were screened in vitro to assess their anti-leukemic and anti-LSC effects. Using this approach, we identified several compounds that display anti-LSC activity in vitro.

## Materials and methods

### AML and normal hematopoietic cell specimens

All AML specimens used in this study were from adult patients at the University Health Network (Toronto, Canada). Human cord blood cells from full-term deliveries were provided from consenting healthy donors at the Cord Blood Bank of Centre hospitalier universitaire Sainte-Justine (Montréal, Canada). Briefly, mononuclear cells were isolated using Ficoll (GE Healthcare) and were enriched 60–90% for CD34+ hematopoietic and progenitor cells (HSPCs) (STEM CELL Technologies). Informed consent was obtained according to the procedures approved by the Research Ethics Board of the respective institutions. Collection of AML samples and mononuclear cell isolation have been previously described^19^.

### In silico analysis

The list of drug candidates was obtained by querying the Connectivity Map database (CMap build 02) with both LSC-R (Eppert et al.^19^) and an additional signature (LSC-Ng) derived from 78 additional AML (Ng et al.^20^) as well as HSC-R (Eppert et al.^19^). The LSC-R up and down probes sets are the 100 probes most significantly correlated with LSCs and 100 probes most correlated with non-LSCs from Eppert et al.^19^. The LSC-Ng probes sets are derived from an early build of the LSC expression data from Ng et al.^20^. The top and bottom 250 genes associated with LSCs (determined by *t*-statistic) were converted to Affymetrix probe IDs (Supplementary Table 1). The HSC-R probes lists are derived from the list of probes associated with HSCs (top and bottom) with a *p* value of ≤0.05. The molecules displaying a negative mean enrichment score (ES) with a *p* value of ≤0.1 for the LSC signatures and that were not associated with a negative ES in HSC-R were considered for in vitro screening.

### Cell culture

Primary AML and cord blood samples were cultured using StemSpan^TM^ SFEM II (STEMCELL Technologies) with growth factors (Life Technologies) (AMLs: 10 ng/mL interleukin (IL)-3, IL-6 and granulocyte colony-stimulating factor (G-CSF), 25 ng/mL thrombopoietin (TPO), 50 ng/mL stem cell factor (SCF) and FLT3 ligand (FLT3L); cord blood: 10 ng/mL IL-6 and G-CSF, 100 ng/mL SCF, FLT3L and 15 ng/mL TPO), and penicillin–streptomycin (Life Technologies). Then, 500 nM of SR1 was included in the culture media for AMLs 9706 and 9642. The MOLM-13 cell line was obtained and cultured per the specification of Deutsche Sammlung von Mikroorganismen und Zellkulturen (DSMZ). AML 8227 was cultured for up to 16 weeks under the same conditions as other primary AMLs described above^23^. All cells were incubated at 37 °C with 5% CO_2_.

### In vitro assay to assess effect of compounds on AML and cord blood

Compounds were purchased from Tocris Bioscience, Cedarlane or Sigma-Aldrich. Primary AML cells or CD34+ enriched human cord blood cells were plated as described above. Candidate molecules or dimethyl sulfoxide (DMSO; Fisher Scientific) were added to the cells at specified concentrations and incubated for 6 days for 8227 AML cells and 4 days for primary AML and cord blood samples. Cells were analyzed by flow cytometry. Briefly, for AML cells, phenotype and viability were assessed using CD34-APC or APC-Cy7 (581), CD38-PE (HB-7), CD15-FITC (HI98), SYTOX Blue (Life Technologies) and when necessary CD33-APC (WM53) and CD14-AlexaFluor 700 (HCD14). HSC phenotype and viability were assessed using CD34-APC-Cy7, CD33-APC, CD38-PE, CD19-PerCP-Cy5.5 (HIB19), CD15-FITC and SYTOX Blue (Life Technologies). All antibodies were purchased from Biolegend. Flow cytometry was performed using a LSRFortessa fitted with a high-throughput sampler (BD Biosciences).

### Colony formation assay

Cells were treated with drugs or DMSO as control for 4 days. The same volume of cell suspension was used to perform the assay for each condition as determined by the cell count of DMSO control. Cells were diluted with Iscove's modified Dulbecco's medium (Life Technologies), 2% fetal bovine serum (FBS; Wisent), seeded in MethoCult media (#04435, STEMCELL Technologies) in duplicate. The assay duration was 12 days prior to counting colonies.

### Cell cycle and apoptosis

MOLM-13 cells were grown in serum-free RPMI 1640 medium (Life Technologies) for 24 h followed by 12 h of incubation in medium containing 20% FBS (Wisent) and were then treated with 10 μM astemizole or DMSO. The effect of a 24 h treatment on the cell cycle distribution and late apoptosis was evaluated using the APO-BRDU^TM^ Kit (BD Biosciences). Cells were fixed in 1% (w/v) paraformaldehyde (Electron Microscopy Sciences, Pennsylvania, USA) in phosphate-buffered saline (Life Technologies). Washed cells were suspended in 70% (v/v) ethanol. DNA labeling and staining (FITC-labeled anti-BRDU and propidium iodine/RNase staining buffer) were performed as described by the manufacturer (BD Biosciences). DNA breaks and cell cycle phase distribution were evaluated by flow cytometry. To discriminate between G0/G1, cells were fixed and permeabilized using the BD Cytofix/Cytoperm kit (BD Biosciences). Cells were stained with Ki-67 AlexaFluor 700 (Ki-67) and Hoechst 33342 (ThermoFisher Scientific) and analyzed by flow cytometry.

### Gene set enrichment

Functional enrichment analysis was performed by integrating the astemizole transcriptomic data from CMap. Data rank matrix was exported from CMap and instances of cells treated with astemizole (1365: HL60, 2049: PC3, 4471: PC3, 6807: MCF7 and 2211: MCF7) were extracted and probes converted to gene symbols. The ranked expression of probes was summed by genes and then ordered highest to lowest to perform a gene-set enrichment analysis (GSEA, Broad Institute, CA, USA) using Molecular Signatures Database (MSigDB) Collections (c2.cp.reactome.v6.0.symbols.gmt). The number of permutations was fixed at 1000, maximum size at 1000 and minimum size at 8^24^. The enrichment map was generated from the GSEA above using Cytoscape 3.6.0 and the Enrichment Map and AutoAnnotate apps^25,26^. GSEA analysis of 8227 fractions and the LSC signatures was performed using the control sample data from GSE55814. GEO2R was used to generate a ranked list of LSC-related genes (6 LSC CD34+ CD38− samples vs 12 non-LSC CD34− samples) used in GSEA analysis.

### Statistical analysis

The concentration effect curve graph and the calculation of half lethal concentration (LC_50_) were performed using Prism version 6.00 for Mac, GraphPad Software (California, USA). Data are represented as mean ± s.d. and significant differences (*p* ≤ 0.05) between groups were determined by a two-way unpaired Student’s *t*-test. Flow cytometry data analysis was performed using FlowJo (v X10.1).

## Results

### In silico identification of candidate anti-LSC compounds

**Fig. 1:**
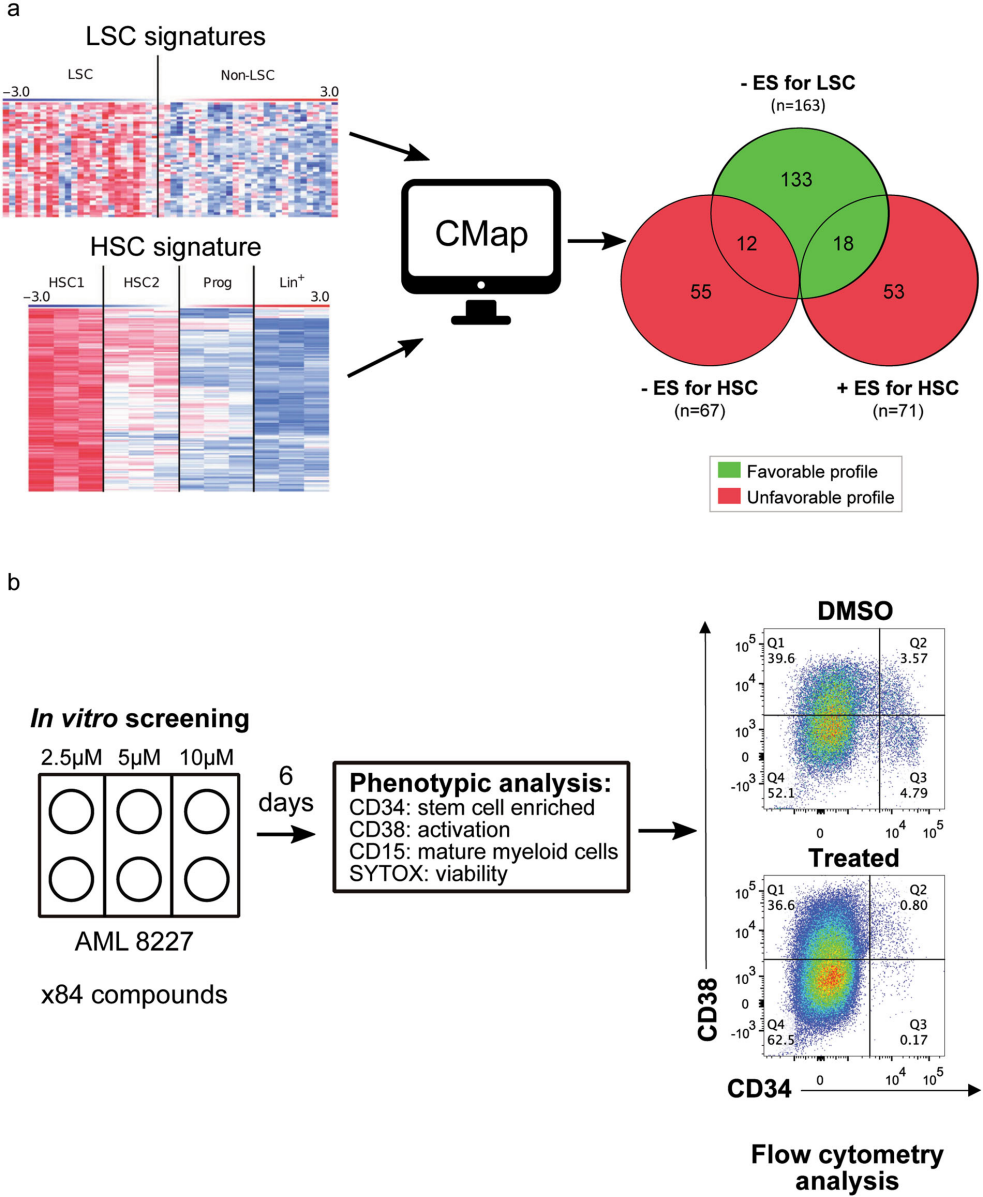
In silico and in vitro screening for drugs preferentially targeting LSCs over HSCs. **a** Schematic illustrating the in silico screen process for identifying anti-LSC compounds. The Venn diagram indicates the number of candidate compounds that target LSCs without harming HSCs (*n* = 133) or enhance the HSC-signature (*n* = 18). The + or – ES indicates positive or negative enrichment score. **b** Experimental design of the in vitro screening and analysis process of 84 candidate compounds

To identify compounds that may specifically target LSCs and spare normal HSCs, we queried the CMap with our established LSC and HSC signatures (Fig. 1a, Supplemental Table 1)^19,20^. CMap contains the expression profiles of multiple cancer cell lines treated with 1310 small bioactive molecules including several well-established FDA-approved drugs. We first queried with our two LSC signatures (LSC-R and LSC-Ng) to identify compounds predicted to inhibit the LSC gene expression programs. We then assayed the CMap data with the HSC-R signature to identify compounds that may inhibit HSC gene programs and filtered the LSC results to remove the former compounds. This resulted in a total of 151 compounds predicted to target LSC-specific gene profiles, including 133 molecules expected to have no effect on HSC-specific gene expression and 18 compounds predicted to enhance the HSC-specific gene profile (Fig. 1a, Supplemental Table 2).

### Evaluation of anti-leukemic activity of in silico candidates: in vitro drug screening

To evaluate our anti-LSC candidate compounds, we used our in vitro AML 8227 model^23^. AML 8227 is an aggressive, relapsed leukemia sample with mutations in *p53*, *RUNX1* and *FLT3-ITD* that maintains a phenotypic and functional LSC hierarchy in culture and can be grown indefinitely (see ref.^23^ and K. Eppert, unpublished data). The cultured LSCs maintain the same LSC gene expression profile as the primary patient LSCs. The LSC signatures are preferentially expressed in 8227 LSCs and it is therefore an excellent model to examine CMap predicted compounds (Supplemental Fig. 1). The LSCs in the primary patient sample and during in vitro culture are CD34+ CD38−, while leukemic progenitors are CD34+ CD38+ and the CD34− cells are terminally differentiated CD15+ blasts. AML 8227 LSCs can be maintained in culture for several weeks without the addition of compounds such as SR1 or UM729 that forcibly inhibit differentiation, thus making it ideal for screening potential anti-LSC compounds^27^. Critically, the unique phenotypic marker profile of the LSCs compared to more differentiated cell populations in 8227 AML facilitates our in vitro screen in which AML 8227 cells are exposed to compounds and the effects on LSCs and blasts are quantifiable by flow cytometry (Fig. 1b).

**Fig. 2:**
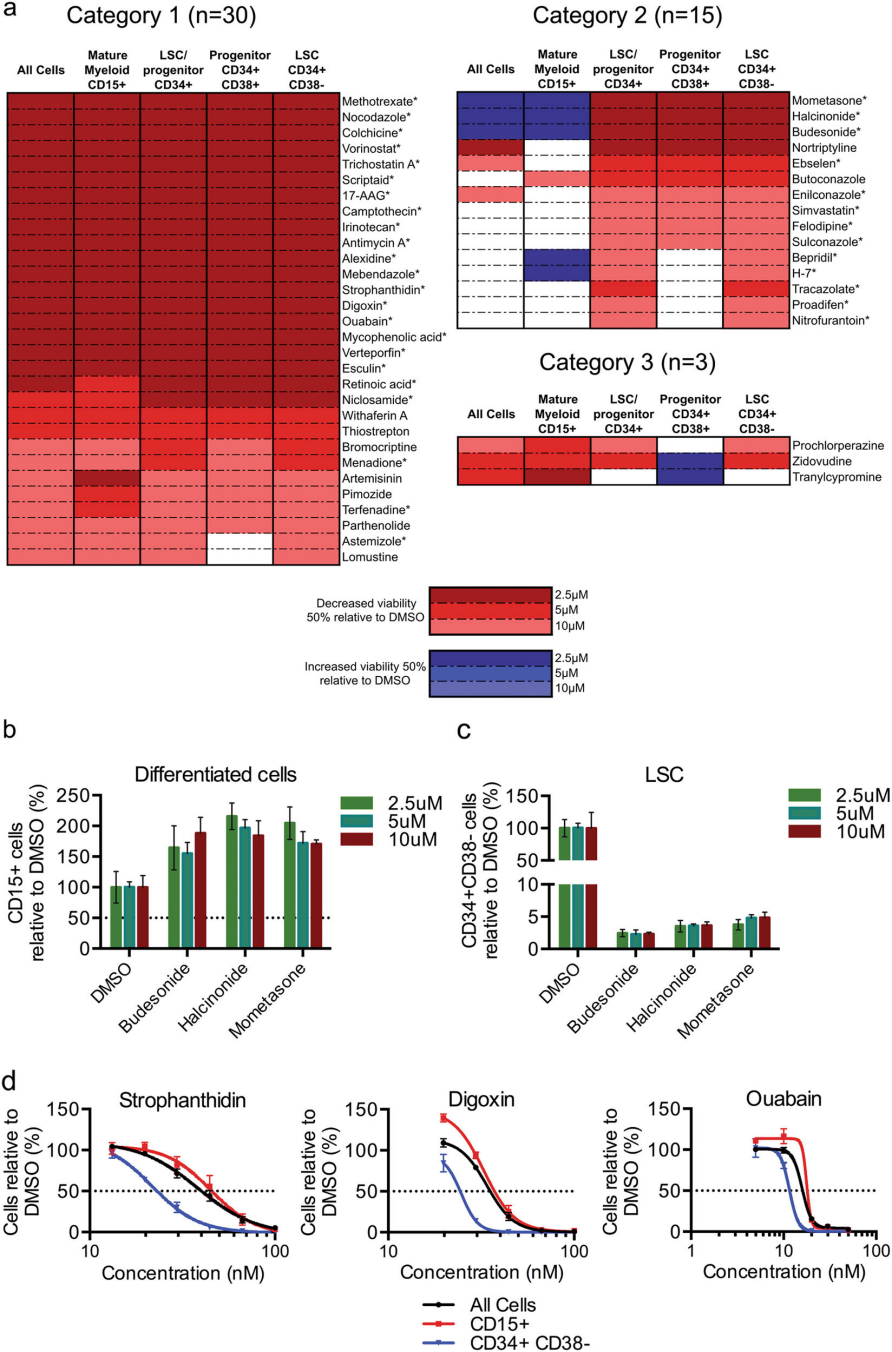
In vitro validation of anti-leukemic compounds from an in silico screen against AML 8227. **a** Summary of all compounds that affected at least one population in 8227 AML cells. Red denotes decreased viability of at least 50% at the indicated concentration and blue denotes increased viability of at least 50% at the indicated concentration; experiment performed in duplicate. An ‘*’ indicates that the compound was retested. **b**, **c** Confirmation of the effect of 9 compounds shown to be preferential for CD34+ cells on **b** the CD15+ terminally differentiated blast population and **c** the CD34+ CD38− LSC-containing population. **d** Viability of bulk and CD34+ CD38− AML 8227 cells treated with the three cardiac glycosides, strophanthidin, digoxin and ouabain at indicated concentrations for 6 days. Data are representative of three independent experiments performed in triplicate and displays the mean ± s.d.

We selected 84 of the 151 compounds identified in our in silico screen for functional validation in 8227 AML cells (Supplemental Table 2). We chose these compounds because many of them are similar subtypes, suggesting they may have a common anti-LSC effect and mechanism (Fig. 2a). AML 8227 was exposed to three doses of each compound (2.5, 5 and 10 μM) in duplicate for 6 days and the impact on cell number was assessed by flow cytometry for CD34+ CD38− LSC-enriched populations, CD34+ CD38+ leukemic progenitors, CD15+ blasts and ‘bulk’ cells (all cells) (Fig. 1b). Of the 84 compounds screened, 48 (57%) of the compounds affected the 8227 AML cell viability (Fig. 2a and Supplemental Table 2). From these screens, we categorized three types of anti-leukemic compounds: (1) those that target all leukemic populations tested (*n* = 30), (2) those that target the LSC-containing population (*n* = 15) with lower effectiveness against non-LSC cells and (3) those that had other targeting patterns (*n* = 3) (Fig. 2a). Generally, these compounds were toxic, although three glucocorticoid compounds triggered differentiation of the CD34+ population with a concurrent expansion of the CD15+ blast population: budesonide, halcinonide and mometasone (Fig. 2b, c). We retested the category 2 compounds and validated the anti-CD34+ targeting of nine of these compounds (Table 1, Fig. 2b, c). We then examined 20 compounds from category 1, which were highly toxic to all populations at the lowest screening doses (2.5 μM), at the nanomolar range to determine their potential population-specific effects. This led to the reclassification of three drugs belonging to the cardiac glycosides therapeutic class to category 2 (Table 1, Fig. 2d). Assessing the different therapeutic families of drugs that were positive in our screen, we identified three with multiple positive hits: H1-antihistamines from category 1 and cardiac glycosides and glucocorticoids from category 2. These therefore represent excellent anti-LSC candidates that we then characterized further.

**Table 1:** Drugs confirmed to be effective against AML 8227

Compound name	Screen category	Class	Approved (e.g. FDA)	Rule of 5
Astemizole	1	Anti-histamine	Yes^a^	No
Terfenadine	1	Anti-histamine	Yes^a^	No
Simvastatin	2	Lipid-lowering	Yes	Yes
Sulconazole	2	Anti-infective	Yes	Yes
Budesonide	2	Corticosteroid	Yes	Yes
Halcinonide	2	Corticosteroid	Yes	Yes
Mometasone	2	Corticosteroid	Yes	Yes
Menadione	2	Vitamin	Yes^b^	Yes
Ebselen	2	Other	No	Yes
H-7	2	Other	No	Not available
Proadifen	2	Other	No	Yes
Digoxin	2	Cardio-vascular	Yes	Yes
Strophanthidin	2	Cardio-vascular	No	Yes
Ouabain	2	Cardio-vascular	No	Yes

^a^Withdrawn from the market

^b^Discontinued from the market

### H1-antihistamines: astemizole and terfenadine

**Fig. 3:**
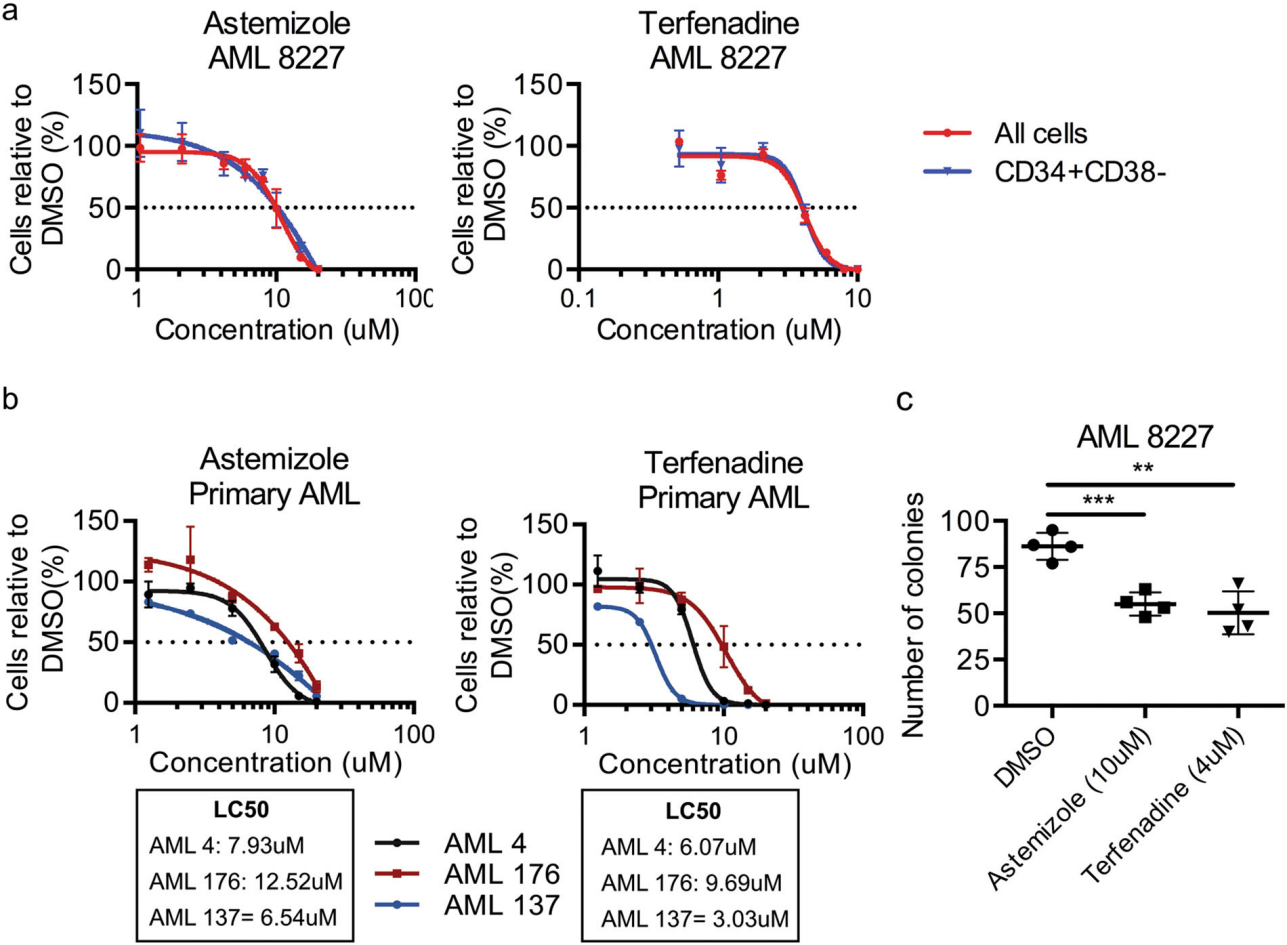
The H1-antihistamines astemizole and terfenadine have anti-leukemic properties against all cells within the AML hierarchy. **a** Viability of bulk and CD34+ CD38− AML 8227 cells treated with astemizole and terfenadine at indicated concentrations for 6 days. Data are representative of three independent experiments performed in triplicate and displays the mean ± s.d. **b** Viability of 3 additional primary AML samples (AML 4, AML 176 and AML 137) treated with astemizole and terfenadine at indicated concentrations for 4 days. Data represent the mean ± s.d.; experiment performed in triplicate. **c** Colony formation assay of AML 8227 after 4 days of treatment with 10 μM astemizole and 4 μM terfenadine. Data are representative of three independent experiments performed in quadruplicate and display mean ± s.d.; *p ≤ 0.05, **p ≤ 0.01, ***p ≤ 0.001, ****p ≤ 0.0001

Astemizole and terfenadine affected all the leukemic populations equally in the micromolar range in the screen against 8227 AML cells (Fig. 2a). These compounds are both inverse agonists of the histamine receptor H1 (HRH1). In total, 23 of the 151 compounds predicted to be effective against LSC in our in silico analysis are known to target HRH1, including anti-allergy drugs, tricyclic antidepressants and antipsychotic drugs. We therefore sought to further investigate astemizole and terfenadine activity against LSCs and the involvement of the HRH1 as a potential mechanism to explain their anti-leukemic activity. Dose response curves revealed an LC_50_ in CD34+ CD38− of 10.21 μM and 4.06 μM for astemizole and terfenadine, respectively, with equal efficacy against the LSC population (CD34+ CD38−) and the total leukemic cell population (Fig. 3a). We then validated the effectiveness of these two compounds against three additional primary human AML samples and observed a similar LC_50_ for each AML (Fig. 3b and Supplemental Table 3). To test whether the compounds were targeting functional primitive cells in these populations we performed colony formation assays that included these drugs. Treatment of AML 8227 with 10 μM astemizole, 4 μM terfenadine or DMSO control led to approximately 50% fewer colonies with drugs compared to control, indicating that the compounds eliminate functional progenitor cells (Fig. 3c).

**Fig. 4:**
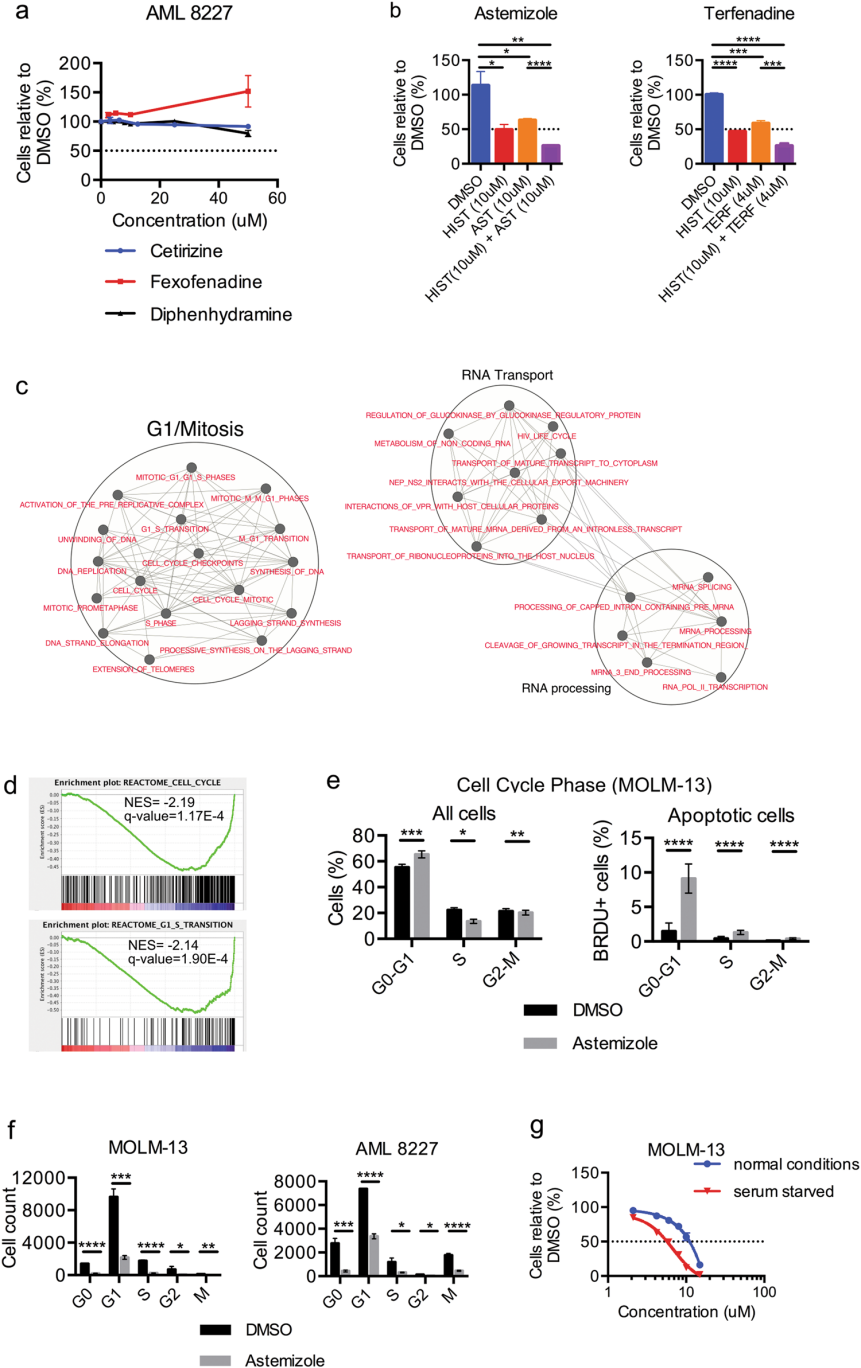
Astemizole initiates apoptosis in the G0/G1 cell cycle phase independently of the H1 receptor. **a** Viability of AML 8227 cells treated with the H1-antihistamines cetirizine, fexofenadine and diphenhydramine up to 50 μM for 6 days. **b** Viability of AML 8227 after the addition of 10 μM histamine to 10 μM astemizole or 4 μM terfenadine treatment for 4 days. **c** Enrichment map of CMap expression data revealing astemizole modulated pathways. Nodes (circles) represent gene sets and edges (lines) represent shared genes. **d** GSEA plot of negative enrichment of cell cycle and G1/S transition using the gene expression signature of cells treated with astemizole from CMap. **e** Apo-BrdU TUNEL assay on MOLM-13 cells treated with 10 μM astemizole for 24 h. **f** Cell cycle analysis using Ki-67 on MOLM-13 and 8227 cells with 10 μM astemizole for 24 h. **g** Viability of MOLM-13 cell growth in serum-starved (0% FBS) or normal conditions (20% FBS) after 24 h of treatment with indicated doses of astemizole. Data are representative of at least two independent experiments performed in triplicate and represent the mean ± s.d.; **p ≤ 0.01, ***p ≤ 0.001

Next, we examined the target pathway for astemizole and terfenadine. We assayed three additional compounds commercialized for their anti-HRH1 activity but did not observe a significant effect on cell viability. Even at concentrations up to 50 μM, fexofenadine (an active moiety of terfenadine), diphenhydramine and cetirizine do not significantly alter the AML 8227 cell count compared to DMSO (Fig. 4a). While all these compounds target the HRH1, these three have a different assortment of additional targets than astemizole and terfenadine. This indicates that HRH1 is not the sole target of the anti-LSC activity of these two compounds. To confirm this, we tested whether the addition of histamine, the endogenous substrate of HRH1, can reverse the phenotype of cell death induced by astemizole and terfenadine. We co-incubated 8227 AML cells with 10μM histamine and either astemizole, terfenadine or control (DMSO) and did not observe a rescue of cell viability but rather histamine has an additive impact to the toxicity (Fig. 4b). Thus, the HRH1 is not the target of astemizole and terfenadine anti-LSC activity.

To determine the mechanism of action of these compounds in AML we performed GSEA using the gene expression signatures of cells treated with astemizole from CMap. This revealed enrichment for gene sets associated with cell cycle and DNA processing and repair, particularly G1/S transition (Fig. 4c, d). To confirm this, we treated the MOLM-13 AML cell line with astemizole or DMSO for 24 h and examined cell cycle distribution and apoptosis. We observed an apparent inhibition of the cell cycle in the G0/G1 phase (G0/G1 = + 6.7%, S = −2.6% and G2M = −3.9%, *p* ≤ 0.01); this inhibition was associated with a higher level of apoptosis compared to other cycle phases (G0/G1 = + 7.6%, S = + 0.9% and G2/M = + 0.3%, *p* ≤ 0.002) (Fig. 4e). To determine if astemizole was targeting cells in G0 or G1, we performed further cell cycle analysis using Ki-67. Astemizole eliminated cells in both phases in MOLM-13 and 8227 AML cells (Fig. 4f). Furthermore, serum-starved non-cycling MOLM-13 cells were more sensitive to astemizole compared to cells in normal conditions (LC_50_ = 5.71 µM vs LC_50_ = 11.06 µM) (Fig. 4g), indicating that astemizole can successfully eliminate cells in both G0 and G1.

### Cardiac glycosides target AML primitive cells

**Fig. 5:**
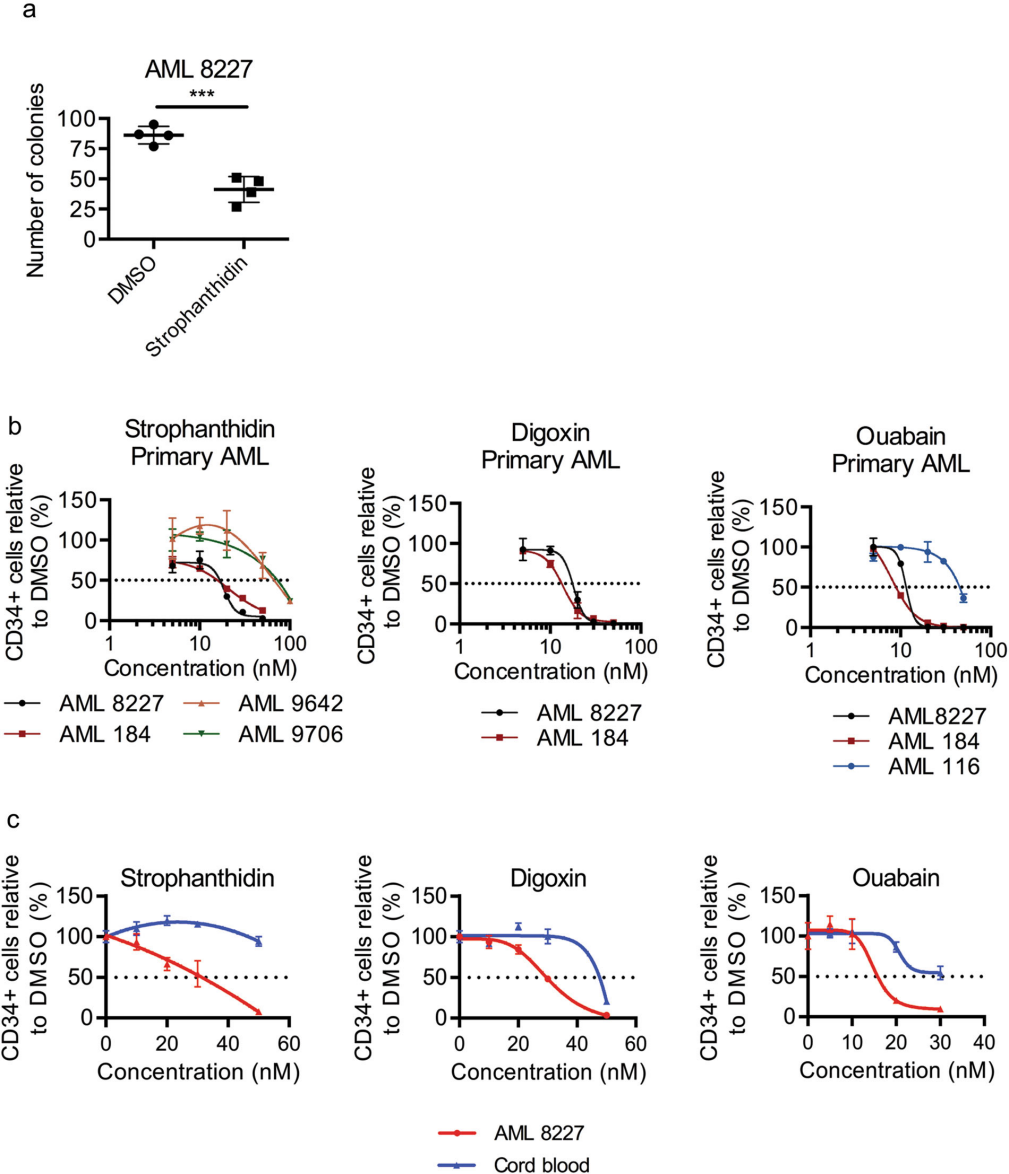
The cardiac glycosides strophanthidin, digoxin and ouabain are cytotoxic against primitive AML cells. **a** Colony formation of AML 8227 treated with 30 nM of strophanthidin for 4 days. Data are representative of two independent experiments performed in quadruplicate and represents mean ± s.d. **b** Viability of additional primary AML treated with strophanthidin, digoxin and ouabain at indicated concentrations for 4 days. Data represent mean ± s.d; experiment performed in triplicate. **c** Viability of CD34+ cord blood cells and CD34+ 8227 cells treated with strophanthidin, digoxin and ouabain at indicated concentrations for 4 days. Data are representative of at least two independent experiments performed in triplicate and represent the mean ± s.d. ***p ≤ 0.001

As shown during our initial screen, the cardiac glycosides strophanthidin, digoxin and ouabain are effective against 8227 AML cells in the nanomolar range, with high toxicity towards CD34+ CD38− cells (LC_50_: strophanthidin = 23.25 nM, digoxin = 23.19 nM, ouabain = 11.61 nM) (Fig. 2d). A colony formation assay using AML 8227 treated with strophanthidin resulted in significantly fewer colonies compared to the DMSO control, supporting the ability of cardiac glycosides to eliminate functional leukemic progenitor cells (Fig. 5a). To assess efficacy against primitive cells in primary AML, we tested the response of CD34+ cells to treatment in multiple primary human AML samples (Supplemental Table 3). We observed two patterns of response: AML 8227 and AML 184 were sensitive to the cardiac glycosides at low nanomolar doses (LC_50_: 8.90–25.55 nM), while AML 116, AML 9642 and AML 9706 were killed at higher doses (LC_50_: 33.73–77.04 nM) (Fig. 5b). Sensitivity or resistance to cardiac glycosides is likely determined by underlying genetic/epigenetic differences between the AML.

The in silico analysis of LSC and HSC signatures predicted that the cardiac glycosides would be less toxic against normal HSCs than AML cells. We therefore assessed the toxicity of all three cardiac glycosides against normal hematopoietic cells using CD34+ enriched normal human cord blood samples, a rich source of HSPCs. All three drugs are less toxic against CD34+ HSPC cord blood cells compared to CD34+ 8227 AML (LC_50_ for AML 8227 and cord blood were respectively: strophanthidin = 29.59 nM and 68.41 nM; digoxin = 29.57 nM and 43.40 nM and ouabain = 15.17 nM and 31.45 nM) (Fig. 5c).

### Glucocorticoids eliminate LSCs by differentiation

**Fig. 6:**
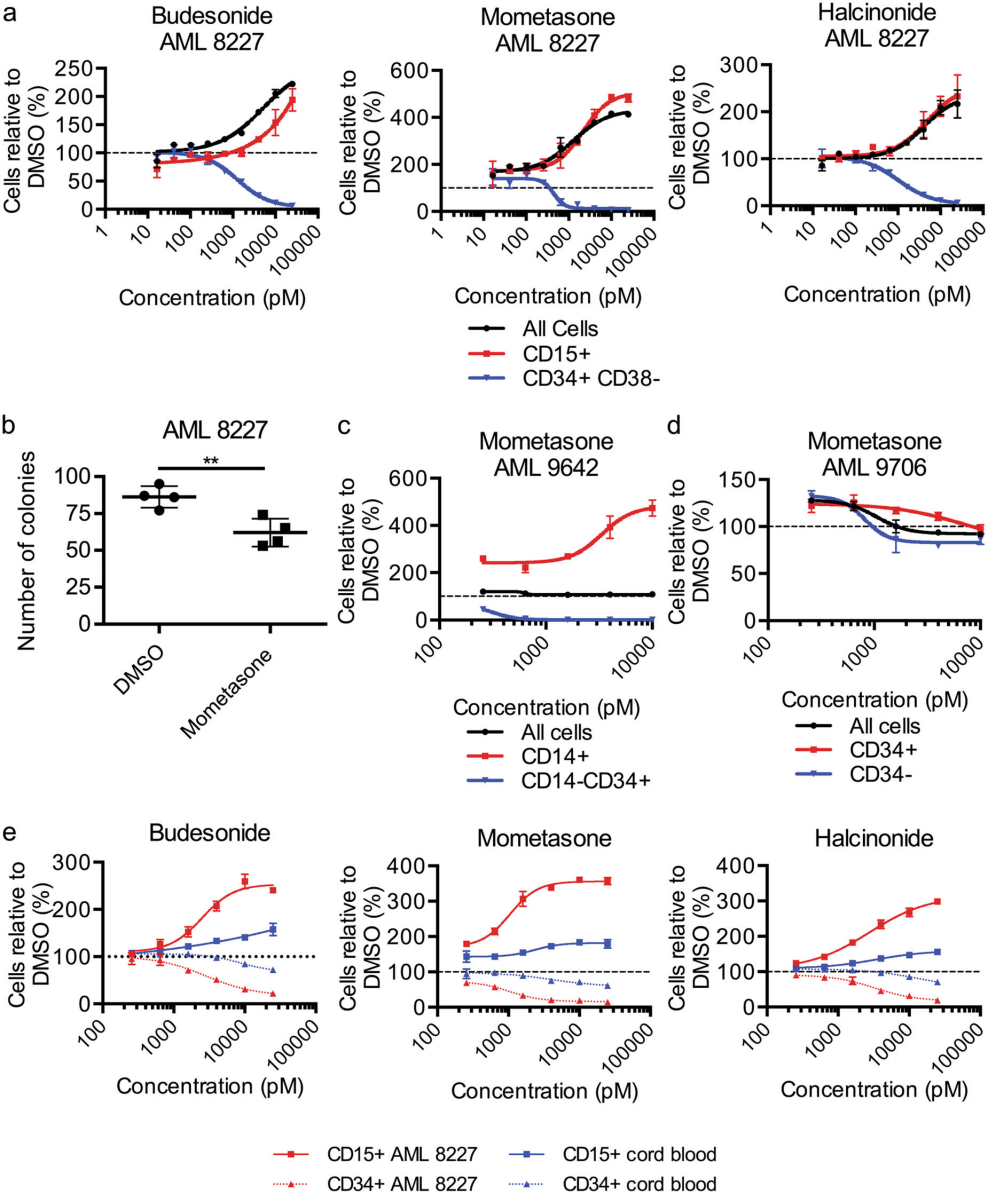
The glucocorticoids budesonide, mometasone and halcinonide differentiate AML samples with limited toxicity to HSPCs. **a** Viability of bulk, CD15+ (mature blast) and CD34+ CD38− AML 8227 cells treated with budesonide, mometasone and halcinonide at indicated concentrations for 6 days. Data are representative of at least three independent experiments performed in triplicate and represents the mean ± s.d. **b** Colony formation of AML 8227 treated with 1 nM of mometasone for 4 days. Data are representative of two independent experiments performed in duplicate and represents mean ± s.d. **c**, **d** Viability of **c** bulk, CD14+ (mature blast) and CD14– CD34+ AML 9642 cells and **d** bulk, CD34– and CD34+ AML 9706 cells treated with indicated concentrations of mometasone for 4 days. Data represent mean ± s.d; experiment performed in triplicate. **e** Viability of CD34+ and CD15+ mature cord blood cells, as well as CD34+ and CD15+ AML 8227 cells treated with budesonide, mometasone and halcinonide at indicated concentrations for 4 days. Data are representative of two independent experiments performed in triplicate and represents the mean ± s.d.; **p ≤ 0.01, ***p ≤ 0.001

We observed that the compounds budesonide, mometasone and halcinonide drastically differentiate the CD34+ primitive cells to CD15+ terminally differentiated blasts (Fig. 2a–c). These three compounds are all part of the glucocorticoid class of drugs. When tested at lower concentration, we determined that all three corticosteroids are effective in the low nanomolar range against 8227 AML CD34+ CD38− cells (LC_50_ budesonide = 1.41 nM, mometasone = 0.57 nM, halcinonide = 1.20 nM) (Fig. 6a). To confirm that these corticosteroid compounds eliminate primitive cells we performed a colony formation assay and observed that mometasone successfully eliminates functional leukemic progenitor cells (Fig. 6b). Furthermore, 8227 cell morphology supports this with increased incidence of condensed chromatin, granularity, heterogeneity and small cells and a corresponding decrease in nucleoli, indicating greater differentiation (Supplementary Figure 2). Next, we tested the activity of the glucocorticoids in primary human AML samples (Supplemental Table 3). We assayed mometasone, the most potent candidate, against two AMLs. AML 9642, which was resistant to the cardiac glycosides, was sensitive to mometasone, while AML 9706 was resistant (Fig. 6c, d).

To investigate whether the glucocorticoids might differentiate HSPCs, we counter-screened all three steroids against CD34+ enriched normal human cord blood samples at doses shown to be effective against AML cells. All three drugs displayed lower toxicities against CD34+ HSPCs, shown by a minimal loss of CD34+ cells and expansion of differentiated CD15+ myeloid cells, than against AML cells (CD34+ LC_50_ for AML 8227 and cord blood: Budesonide = 4.04 nM, and 69.95 nM; Mometasone = 0.86 nM and 50.76 nM and Halcinonide = 3.81 nM and 68.28 nM, Fig. 6e).

### Candidate compound effectiveness in combination with cytarabine

**Fig. 7:**
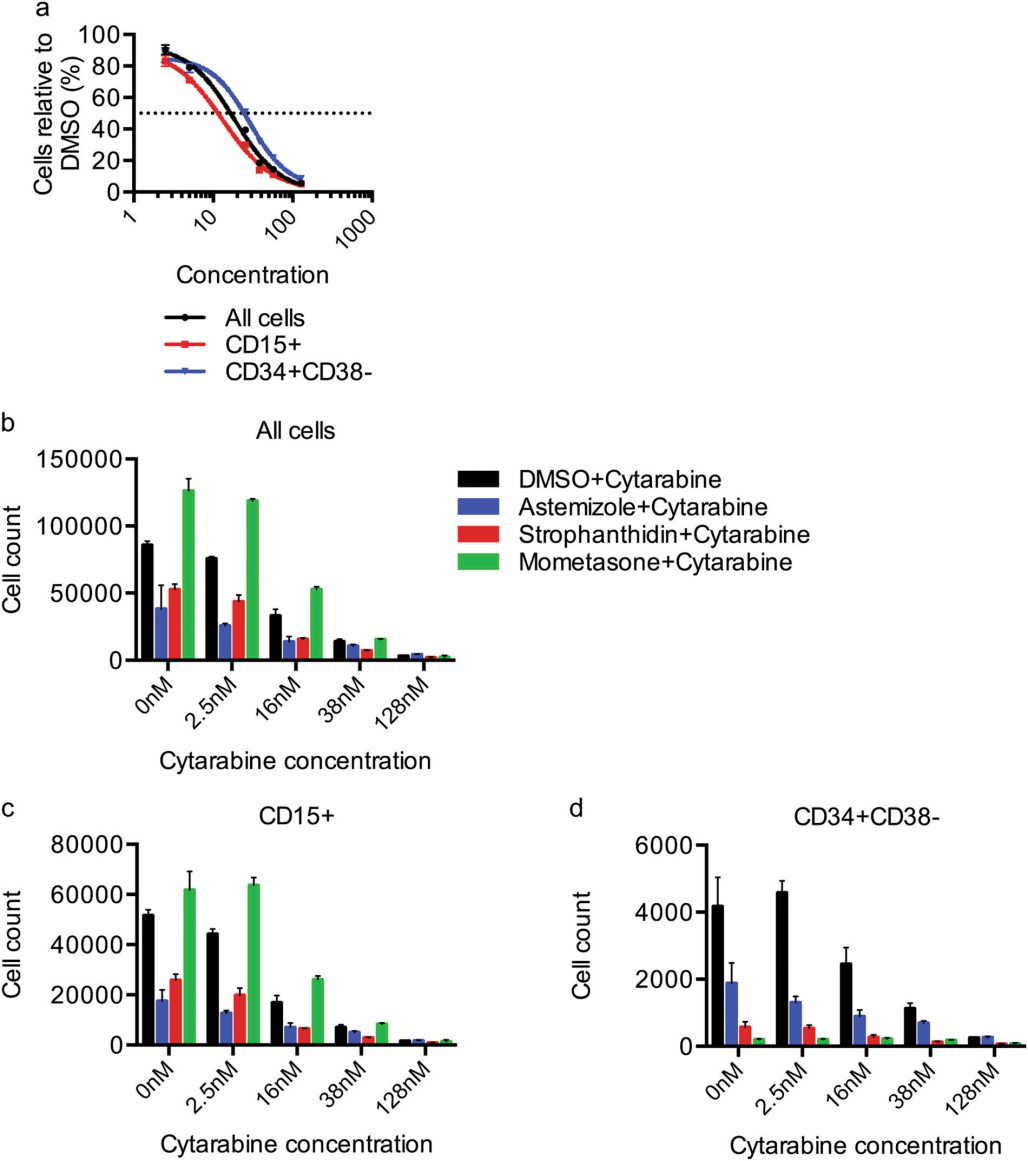
Candidate compounds potentiate the effect of cytarabine in AML 8227. **a** Viability of bulk, CD15+ (mature blast) and CD34+ CD38− AML 8227 cells treated with cytarabine at indicated concentrations for 6 days. **b**–**d** Viability of **b** bulk, **c** CD15+ and **d** CD34+ CD38− AML 8227 treated with cytarabine in combination with either DMSO, 10 µM astemizole, 30 nM strophanthidin or 10 nM mometasone for 6 days. Data are representative of three independent experiments performed in triplicate and represent the mean ± s.d.

To assess the three candidate classes compared to a standard-of-care drug, we determined the LC_50_ of cytarabine for AML 8227 subpopulations. The LSC-enriched fraction was less sensitive to cytarabine than the cells in the non-LSC population (LC_50_: bulk cells = 16.89 nM, CD15+ = 12.31 nM, and CD34+ CD38− = 23.03 nM) (Fig. 7a). We further investigated the effect of the combination of cytarabine with the different drug classes identified (astemizole, strophanthidin and mometasone). The candidate compounds had an additive effect in all populations with cytarabine, suggesting they act through different mechanisms and therefore potentially could be used in combination to potentiate the effect of the gold standard chemotherapy treatment (Fig. 7b–d).

## Discussion

We demonstrate that querying CMap with functionally validated LSC and HSC signatures can identify relevant candidates to be repositioned for use in AML in a timely and economic manner. Indeed, from more than 1000 small bioactive molecules listed in CMap, the cross-comparison of drug lists identified using both LSC and HSC signatures reduced the potential candidates by nearly 10-fold. Over half of the drugs identified from this approach successfully demonstrated activity against AML in vitro. CMap includes well-established Food and Drug Administration (FDA)-approved drugs and 9/14 of our primary candidates have been approved in a jurisdiction for human use^28^. This is advantageous because the translation of these drugs to clinical use is substantially less difficult compared to a novel compound that has never been clinically tested^6,7^. A critical feature of our strategy is to use a cell system that enables the analysis of LSC populations in vitro. This allows for the dissection of the effect of the drugs on bulk cells, CD15+ blasts, CD34+ leukemic progenitors and the LSC-containing population in a screen. As we hypothesized, we identified drugs that act against LSC-enriched fractions and, in some cases, all cell populations tested.

We first focused on a group of positive hits comprising drugs sharing an H1-antihistaminine activity, which could potentially be the common mechanism targeting the leukemic cells. In particular, we observed that astemizole and terfenadine, both commercialized for their action against HRH1, were effective against AML LSC and bulk cell populations. There is growing evidence supporting the therapeutic potential of these two drugs as well as other H1-antihistamines for a variety of malignancies, including leukemia, myeloma, breast, prostate, colon, lung and liver cancers^29–35^. The evaluation of three additional molecules in this class, namely diphenhydramine and cetirizine (not predicted to target LSCs in our in silico analysis) and fexofenadine (not included in CMap), did not alter leukemia cell viability even at high micromolar concentrations. This suggests that engagement of HRH1 is not the causal mechanism explaining the action of terfenadine and astemizole on LSCs^36^. This conclusion was further strengthened by the fact that co-treatment of AML cells with histamine together with either astemizole or terfenadine did not reverse the cell death phenotype. Rather, this combination led to potentiation of cell mortality. Similarly, Jakhar et al.^37^ reported that histamine synergizes with the antiproliferative effect of astemizole on MCF7 breast cancer cells. They demonstrated that combined use of these molecules triggered endoplasmic reticulum (ER) stress-induced apoptotic and autophagic cell death by disruption of calcium (Ca^2+^) homeostasis^37^. Furthermore, terfenadine has been shown to induce apoptosis and autophagy by the depletion of ER Ca^2+^ stores and Ca^2+^ influx from extracellular medium in melanoma cells^31,38^. Cell proliferation is another process regulated by Ca2^+^-dependent signaling pathways, including expression of cell cycle regulator controlling of the G1/S cell cycle transition^39^. The H1-antihistamines terfenadine and cyproheptadine were shown to cause G0/G1 cell cycle arrest and apoptosis in leukemia^29,40^. Our gene-set enrichment analysis of transcriptomic fingerprints of astemizole in cancer cells was consistent with a possible alteration of G1/S cell cycle progression. This was further supported by our in vitro results showing an apparent cell cycle arrest in G0/G1 associated with increased apoptosis. The ability of astemizole to cause cell death mainly in G0/G1 phase suggests that this molecule can eradicate quiescent cells, such as LSCs. However, this remains to be further investigated. From a clinical point of view, it could be argued that the repositioning of astemizole and terfenadine would be limited by their potential severe adverse effects^41^. Both drugs were removed from the market following commercialization due to causing prolonged QT time and ventricular tachycardia. Thus, the concentration required to suppress cancer in a patient will need to be clarified with regard to the proarrhythmic risk deemed acceptable to treat a potential life-threatening disease. The combination of low doses of terfenadine or astemizole synergizes with chemotherapy, and this could be a feasible option for their repurposing in clinic since risk of cardiac events is decreased at lower doses^34^. Furthermore, use of cationic amphiphilic drug antihistamines, comprising astemizole and terfenadine, was linked to lower mortality in a large pharmacoepidemiological study of Danish cancer patients, supporting the use of these compounds^32^. Development of novel astemizole or terfenadine-related compounds with higher potency and more favorable toxicity profile is another possible strategy.

Cardiac glycosides of the cardenolide functional class are another class of drugs for which we demonstrated the ability to alter LSC viability in vitro. This finding is in line with the anti-tumor capability reported for cardiac glycosides in multiple tumor types including breast, lung and melanoma, among others^42–44^. These drugs are inhibitors of the Na^+^/K^+^-ATPase pump, which, in addition to transporting ions across the cell membrane, contributes to intracellular signaling involved in cellular functions such as cell proliferation and apoptosis^45–47^. In particular, the binding of cardiac glycosides to the Na^+^/K^+^-ATPase protein was shown to affect intracellular calcium oscillation and the RAS signaling cascade^43,44,48^. Recently, it was elegantly demonstrated that cardiac glycosides can reactivate cancer suppressor genes at least partially through calcium-linked epigenetic regulation, which may participate in the anticancer activity mediated by these drugs^43^. We observed that sensitivity of AML cells toward the cardiac glycosides was 1.5 to 2 times higher compared to normal HSCs under these in vitro conditions, and thus there is the potential that they would preserve, in part, the normal hematopoietic system if used in vivo to treat AML. In support of our findings for cardiac glycosides, ouabain has shown activity against myeloid leukemia cell lines^49,50^, and in vitro effectiveness against primary patient CD34+ AML cells, although the elimination of primary LSCs was not established^50,51^. Furthermore, in support of the use of cardiac glycosides in leukemia treatment, Haux et al.^52^ observed a link between higher digitoxinemia and protection against leukemia/lymphoma in digitoxin users in Norway. Overall, the cardiac glycosides strophanthidin, digoxin and ouabain are likely anti-LSC candidates for further study as treatment options for AML.

The third subset of drugs that we evaluated further were the glucocorticoids that triggered the differentiation of the leukemic progenitor and LSC-enriched CD34+ populations into terminally differentiated CD15+ cells. Differentiation of blasts follows the archetype of all-*trans* retinoic acid (ATRA), the highly successful treatment of acute promyelocytic leukemia (APL) that overrides the block in differentiation of the leukemic blasts^5^. The discovery of ATRA has significantly increased cure rates for APL to over 80%^53^. Furthermore, glucocorticoids, such as prednisone and dexamethasone, are part of the standard of care for acute lymphoblastic leukemia (ALL). They are largely responsible for the survival rates of approximately 90% in pediatric patients as glucocorticoid resistance has been shown to be an adverse prognostic factor in ALL^54,55^. Rather than inducing differentiation, glucocorticoids have been described to trigger apoptosis in ALL^56^. In AML, glucocorticoids induce cell death and differentiation, particularly in chemorefractory disease and RUNX1-mutated AML^57–60^. Dexamethasone was included in older AML treatment regimens, but these had high toxicity, most likely due to the other standard drugs in the combination and the aggressive scheduling^61–63^. Furthermore, dexamethasone and prednisone are included in CMap but were predicted to not be as effective against the LSC signature as our candidate glucocorticoids. Therefore, the three candidates we have identified might offer an improvement over traditionally used glucocorticoids.

Our approach of combining in silico analysis with an in vitro LSC assay can efficiently identify candidate drugs with anti-leukemia properties among therapeutics commonly used for non-malignant diseases. By probing LSC and HSC profiles in CMap we identified positive hits in a variety of therapeutic classes, including H1-antihistamines, cardiac glycosides and glucocorticoids. The ability of a subset of these candidate molecules to differentially decrease cell populations of the leukemic hierarchy was demonstrated using 8227 AML cells. We confirmed drug efficacy in primary, cytogenetically normal AML. Next, further validation in pre-clinical in vitro and in vivo assays using multiple AML subtypes will be needed to determine the efficacy of these drugs across the broad spectrum of AML. The elucidation of the pathways involved in their effects has a great potential for discovery of novel drugable targets. In addition, medicinal chemistry approaches could be utilized to develop analogs with improved anticancer properties and more favorable safety profiles through the modification of their chemical structures. Our approach is not limited to CMap, and could be adapted to query other large-scale datasets of transcriptional profiles of compounds from multiple cell lines to identify additional drugs to be tested. Overall, promising anti-leukemia molecules were identified and further investigations in pre-clinical models are warranted.

## Electronic supplementary material

Supplemental Table 1

Supplemental Table 2

Supplemental Table 3

Supplemental Figure 1

Supplemental Figure 2

Supplementary Legends
