# Supplementary figures and images for: Leukemic stem cell signatures identify novel therapeutics targeting acute myeloid leukemia
Source: Blood Cancer J. 2018 Jun 6;8(6):52. doi: 10.1038/s41408-018-0087-2 (PMC6889502; doi:10.1038/s41408-018-0087-2)

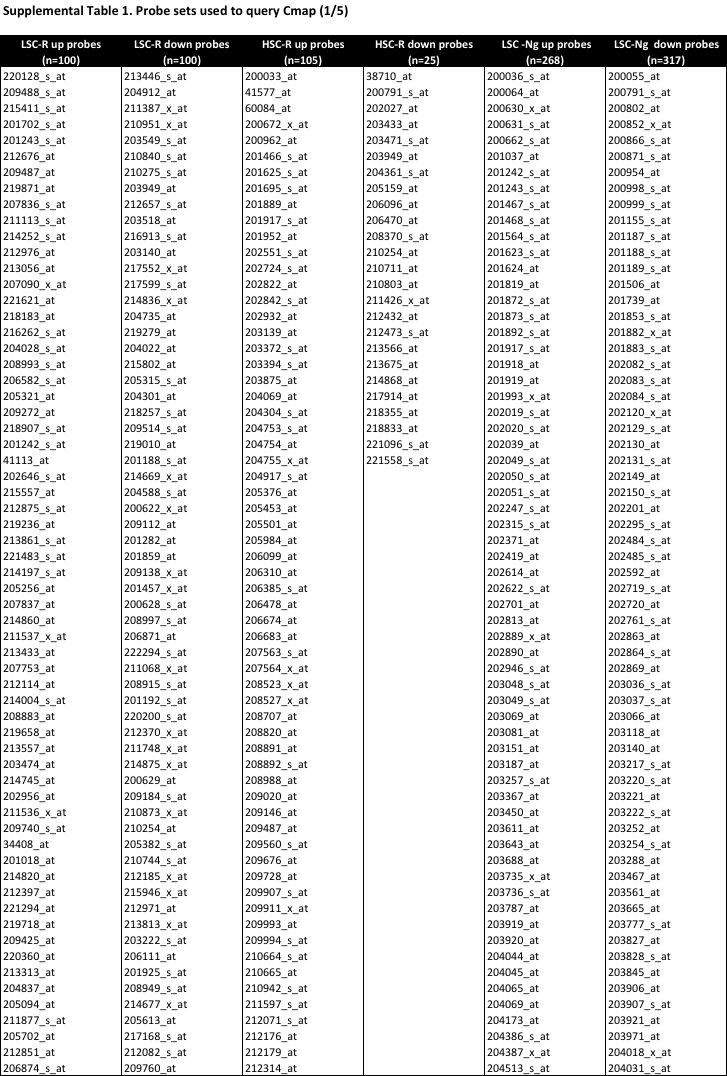


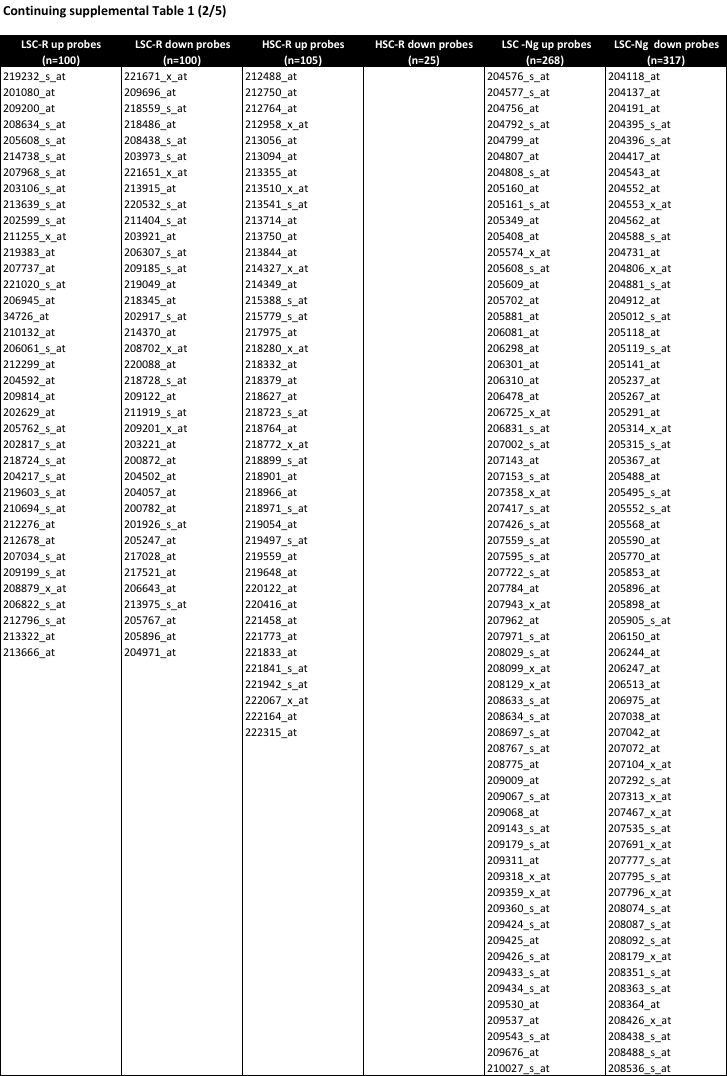


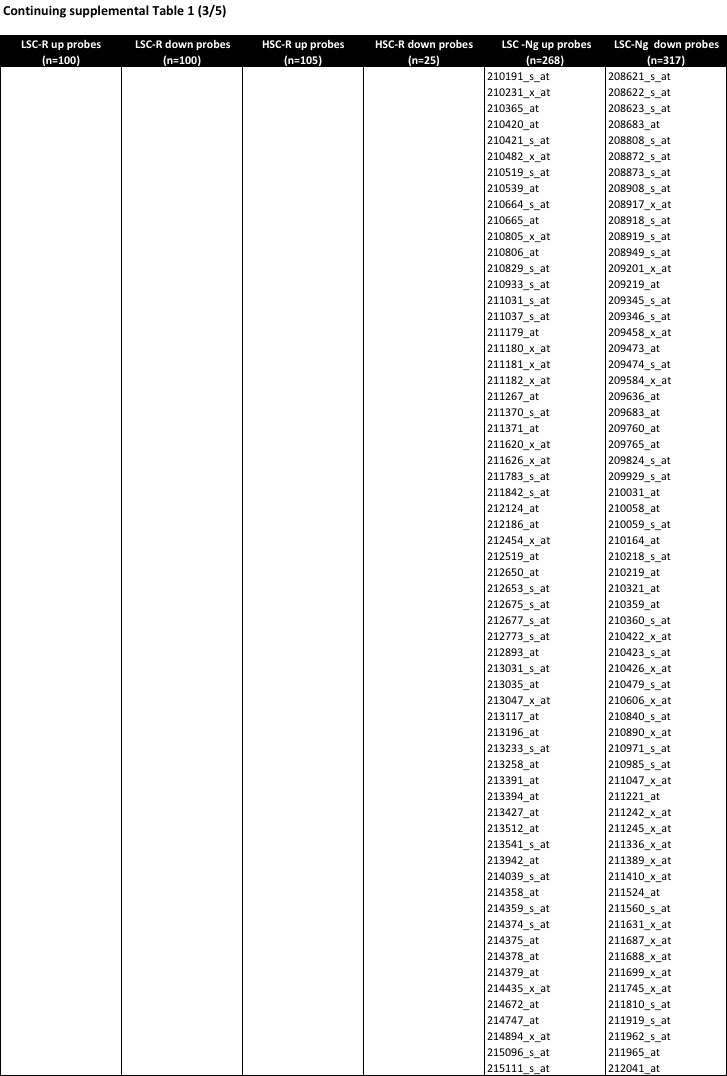


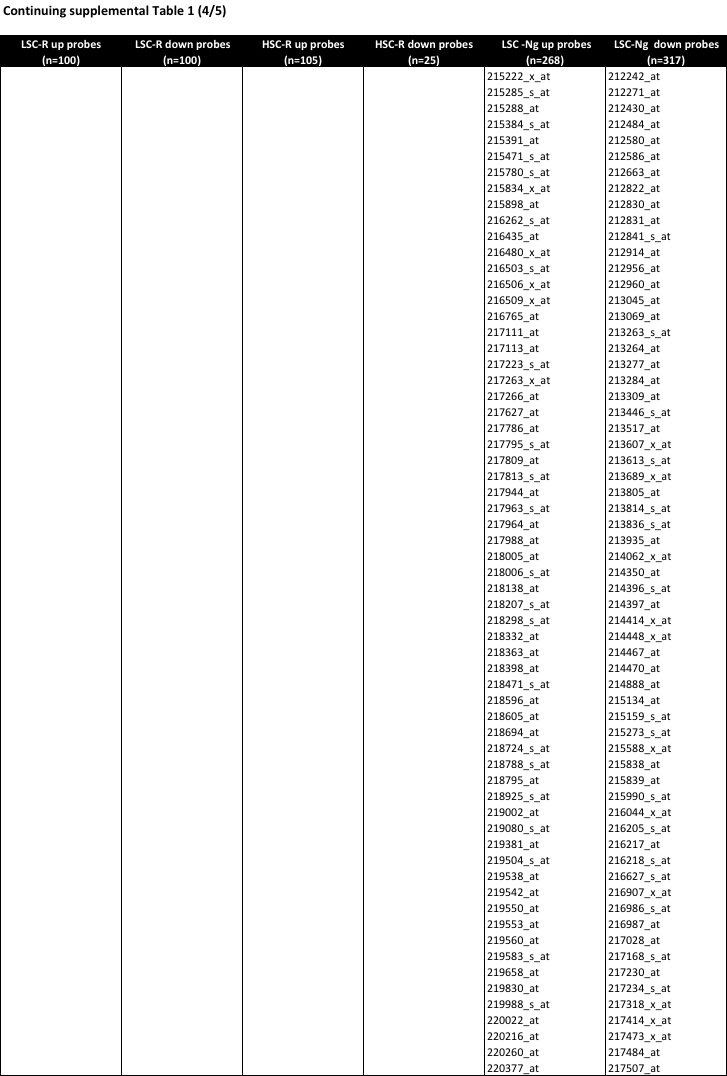


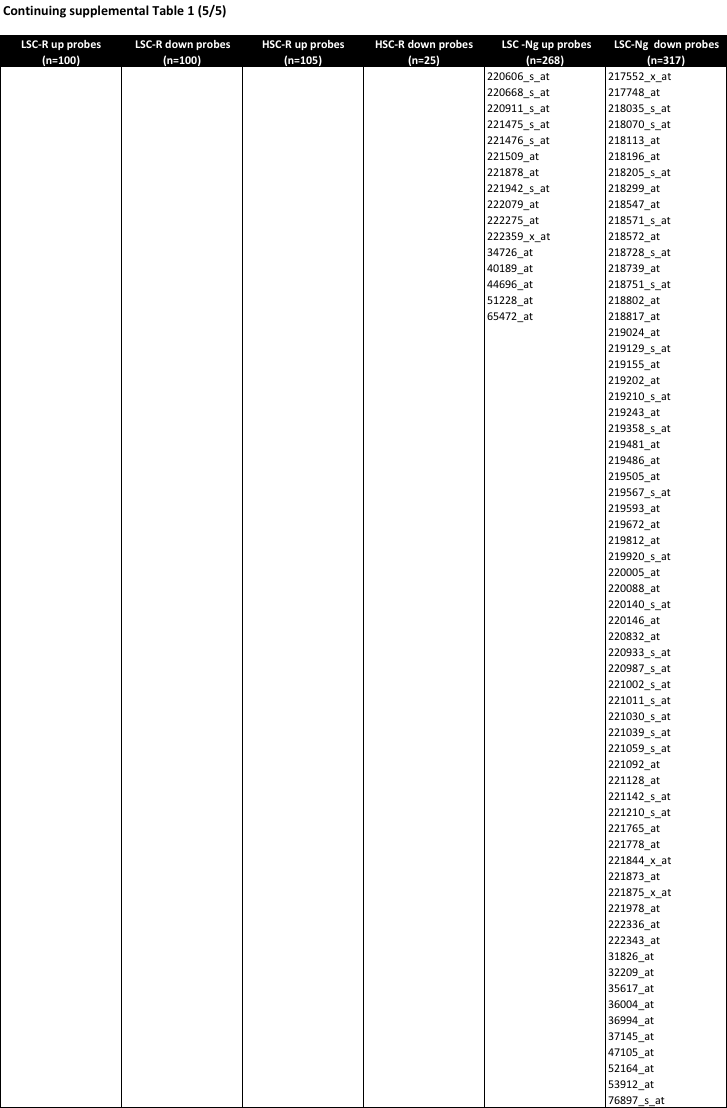

Supplement: Supplementary file 1 — Supplemental Table 1 [file 41408_2018_87_MOESM1_ESM.docx]

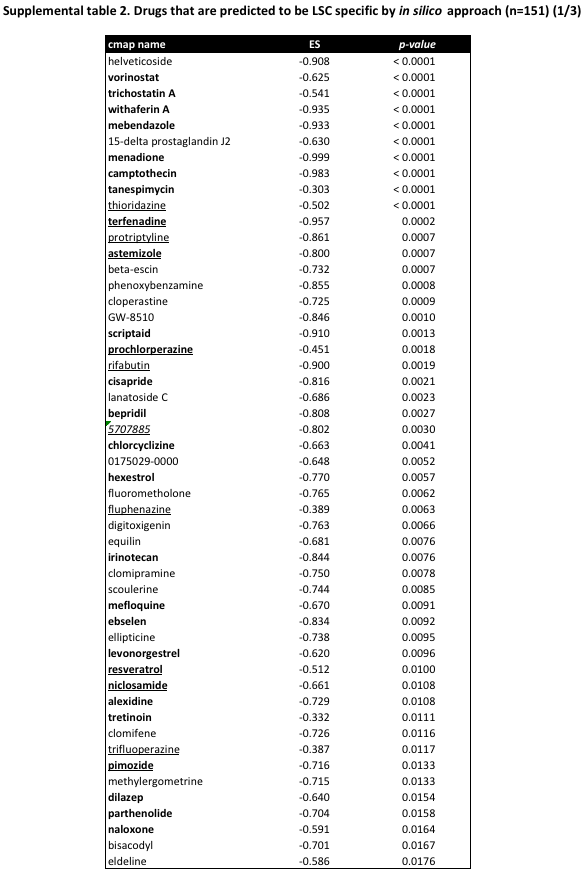


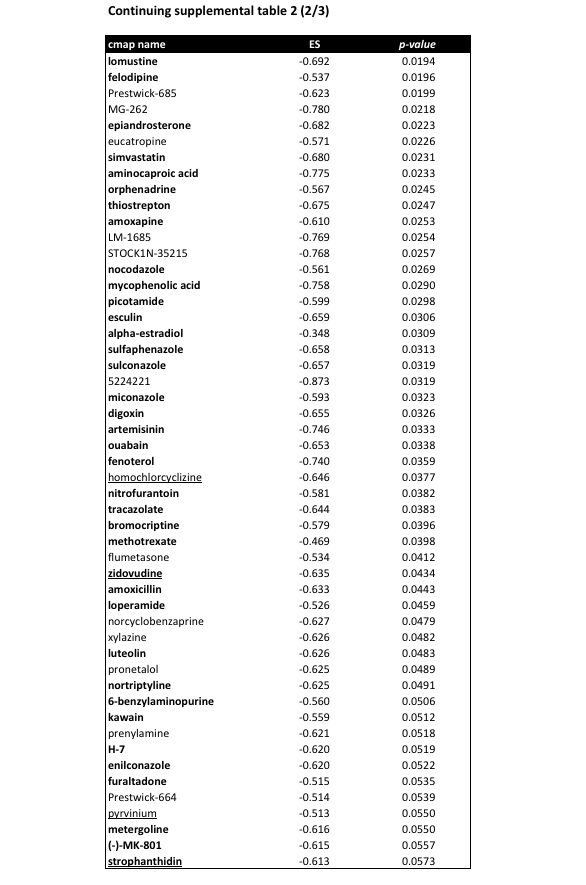


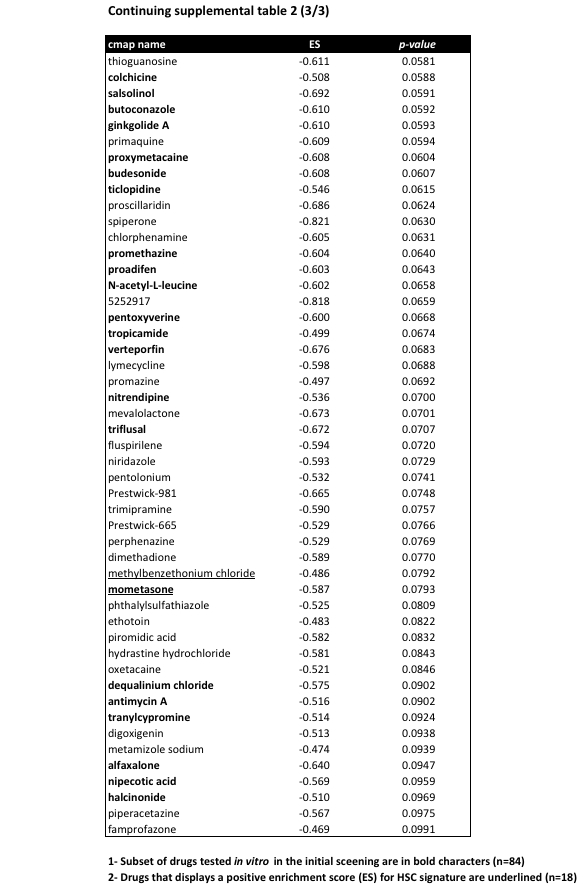

Supplement: Supplementary file 2 — Supplemental Table 2 [file 41408_2018_87_MOESM2_ESM.docx]

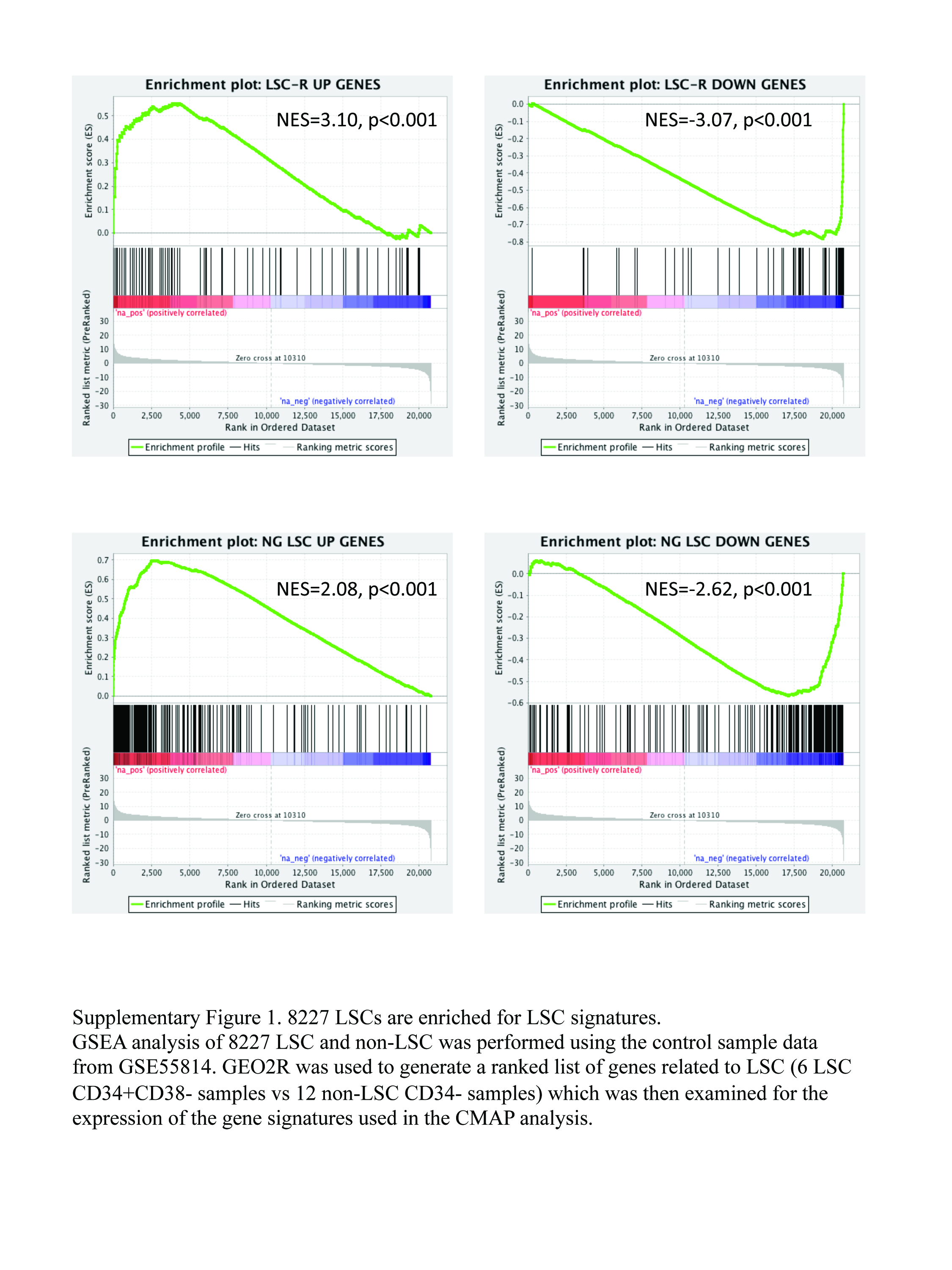

Supplement: Supplementary file 4 — Supplemental Figure 1 [file 41408_2018_87_MOESM4_ESM.tif]

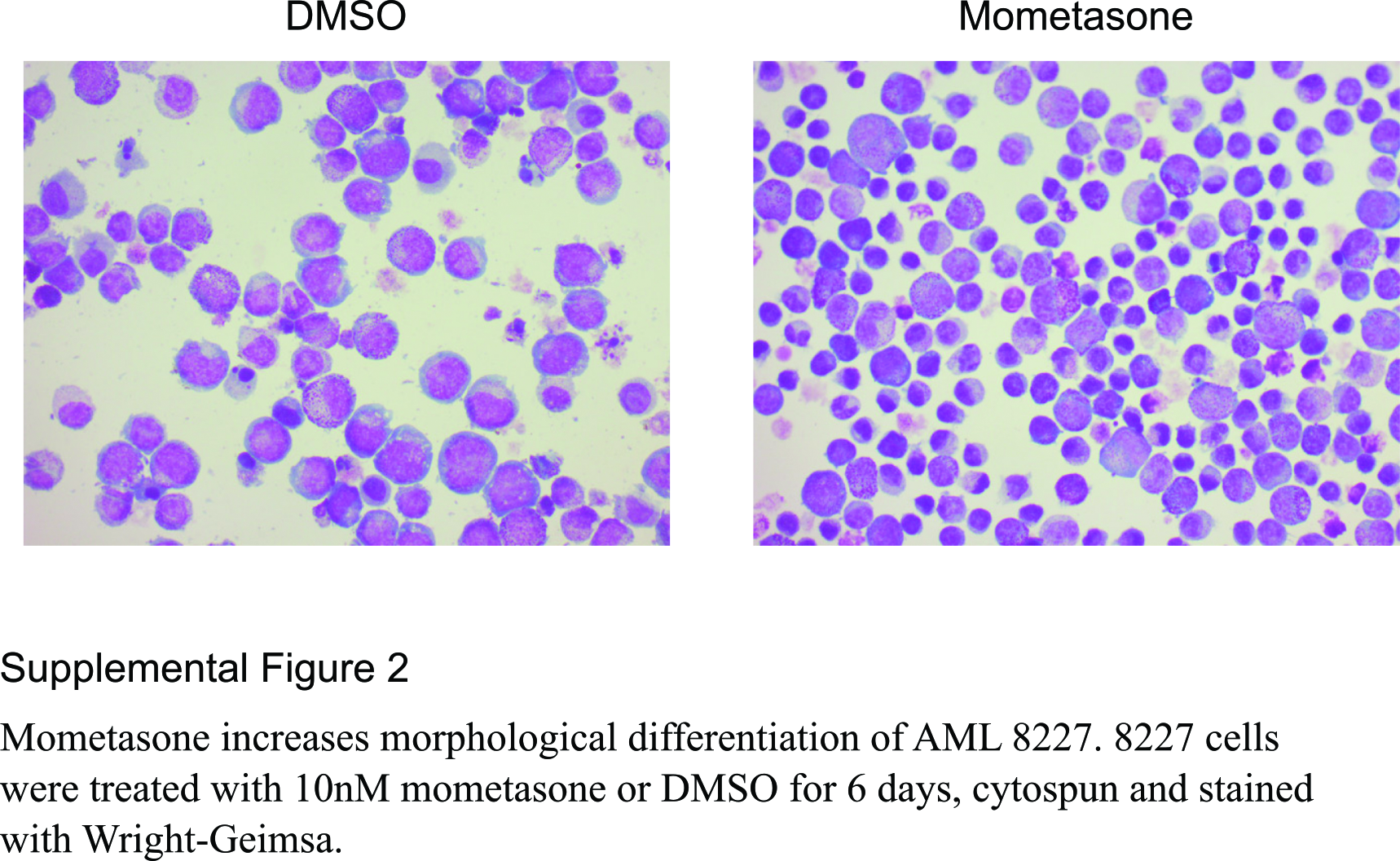

Supplement: Supplementary file 5 — Supplemental Figure 2 [file 41408_2018_87_MOESM5_ESM.tif]
