# Supplementary material for: Leukemic stem cell signatures identify novel therapeutics targeting acute myeloid leukemia
Source: Blood Cancer J. 2018 Jun 6;8(6):52. doi: 10.1038/s41408-018-0087-2 (PMC6889502; doi:10.1038/s41408-018-0087-2)
Supplement: Supplementary file 3 — Supplemental Table 3 [file 41408_2018_87_MOESM3_ESM.pdf]

AML	FAB	NPM1	FLT3	ENL risk
**4**	M1	WT	WT	Intermediate
**116**	M0	WT	WT	Intermediate
**137**	Unknown	WT	WT	Intermediate
**176**	M2	WT	WT	Intermediate
**184**	M4	MUT	TKD	Intermediate
**8227**	M2	WT	ITD	Intermediate
**9642**	M4eo	WT	WT	Intermediate
**9706**	M1	WT	ITD	Intermediate

Supplemental Table 3
