# Supplementary material for: Leukemic stem cell signatures identify novel therapeutics targeting acute myeloid leukemia
Source: Blood Cancer J. 2018 Jun 6;8(6):52. doi: 10.1038/s41408-018-0087-2 (PMC6889502; doi:10.1038/s41408-018-0087-2)
Supplement: Supplementary file 6 — Supplementary Legends [file 41408_2018_87_MOESM6_ESM.docx]

**Supplemental Legends**

**Supplemental Figure 1** 8227 LSC preferentially express LSC-R and LSC-Ng signatures. GSEA analysis of the LSC-enriched (CD34+CD38-) vs non-LSC (CD34-) cell fractions of 8227 using the same LSC gene signatures as used in the CMap analysis.

**Supplemental Figure 2** Mometasone increases morphological differentiation of AML 8227. AML 8227 cells were treated with 10nM mometasone or DMSO for 6 days, cytospun and stained with Wright-Geimsa.

**Supplemental Table 1** Probe sets used to query CMap.

**Supplemental Table 2** Drugs that are predicted to be LSC specific by *in silico* approach (n=451).

**Supplemental Table 3** Primary human AML samples.
